# Zhuifeng Tougu capsules in the treatment of knee osteoarthritis (cold dampness obstruction syndrome): a randomized, double blind, multicenter clinical study

**DOI:** 10.1186/s13020-024-00880-7

**Published:** 2024-01-25

**Authors:** Longmei Zhao, Shasha Zhou, SiWei Wang, Rui Wu, Qingliang Meng, Zhenbin Li, Jiangyun Peng, Ying Liu, Min Lu, Ming Li, Caifeng Zhu, Yue Sun, Yanlin He, Yue Jin, Jingyue Gao, Shumin Zhang, Peihao Li, Rongjun Liao, Wei Liu, Guoming Zhang

**Affiliations:** 1 First Teaching Hospital of Tianjin University of Traditional Chinese Medicine, Tianjin, 300193 China; 2 National Clinical Research Center for Chinese Medicine Acupuncture and Moxibustion, Tianjin, 300381 China; 3 Ordos Traditional Chinese Medicine Hospital, Ordos, 017010 Inner Mongolia China; 4 Hunan University of Chinese Medicine, Changsha, 410208 Hunan China; 5 Hunan Engineering Technology Research Center of Osteoarticular Drugs, Changsha, 410300 Hunan China; 6 The First Affiliated Hospital of Nanchang University, Nanchang, 330006 Jiangxi China; 7 Henan Province Hospital of Traditional Chinese Medicine, Zhengzhou, 450053 Henan China; 8 Bethune International Peace Hospital, People’s Liberation Army, Shijiazhuang, 050082 Hebei China; 9 The First Affiliated Hospital of Yunnan University of Chinese Medicine, Kunming, 650032 Yunnan China; 10 Affiliated Hospital of Shandong University of Traditional Chinese Medicine, Jinan, 250011 Shandong China; 11 The First Hospital of Hunan University of Chinese Medicine, Changsha, 410007 Hunan China; 12 Weifang People’s Hospital, Weifang, 261000 Shandong China; 13 The Second Affiliated Hospital of Anhui University of Chinese Medicine, Hefei, 230061 Anhui China; 14 The First Affiliated Hospital of Anhui University of Chinese Medicine, Hefei, 230031 Anhui China; 15 Tianjin University of Traditional Chinese Medicine, Tianjin, 301617 China; 16 The First Affiliated Hospital of Hunan College of Traditional Chinese Medicine, Zhuzhou, 412008 Hunan China

**Keywords:** Chinese herbal medicine formula, Clinical trial, Knee osteoarthritis, Randomized controlled trial

## Abstract

**Background:**

In Traditional Chinese Medicine (TCM) theory, cold dampness obstruction is one of the common syndromes of osteoarthritis. Therefore, in clinical practice, the main treatment methods are to dispel wind, remove dampness, and dissipate cold, used to treat knee osteoarthritis (KOA). This report describes a mulitercenter clinical study to assess Zhuifeng Tougu Capsule’s efficacy and safety in the treatment of patients who are cold dampness obstruction syndrome in KOA, and to provide evidence-based medical for the rational use of Zhuifeng Tougu Capsules in clinical practice.

**Methods:**

This randomized, parallel group controlled, double-blind, double dummy trial will include a total of 215 KOA patients who meet the study criteria. 215 patients underwent 1:1 randomisation, with 107 cases assigned the experimental group (Zhuifeng Tougu Capsules + Glucosamine Sulfate Capsules Simulator) and 108 assigned the control group (Glucosamine Sulfate Capsules + Zhuifeng Tougu Capsules Simulator). After enrolment, patients received 12 weeks of treatment. The main efficacy measure is the Western Ontario and McMaster University Osteoarthritis Index (WOMAC) pain score. Visual analogue scale (VAS) pain score, Self-condition assessment VAS score, WOMAC KOA score, TCM syndrome score and TCM syndrome efficacy, ESR level, CRP level, suprapatellar bursa effusion depth, use of rescue drugs, and safety indicators are secondary efficacy indicators.

**Results:**

Compared with before treatment, WOMAC pain score, VAS pain score, Self-condition assessment VAS score, WOMAC KOA score, and TCM syndrome score decreased significantly in both groups (*P* < 0.01). Also, the experimental group showed significant differences in the above indicators compared to control (*P* < 0.01). However, after treatment, no significant differences were showed in the ESR level, CRP level, and suprapatellar bursa effusion depth between the two groups (*P* > 0.05). No any serious adverse effects showed in the experimental group and control group.

**Conclusions:**

Zhuifeng Tougu Capsules can effectively improve knee joint function and significantly alleviate the pain of KOA.

*Trial registration*: Clinical trial registration was completed with the China Clinical Trial Registration Center for this research protocol (No. ChiCTR2000028750) on January 2, 2020.

## Introduction

Osteoarthritis (OA) is one of the leading causes of disability in degenerative disease [1]. Its main pathological features are characterized by progressive degeneration of articular cartilage, intra-articular inflammation, cartilage sclerosis, and arthralgia [2], and mechanical stress injury with insufficient joint self-repair is considered a major cause of OA [3]. It is estimated that more than 500 million people are currently affected by osteoarthritis worldwide [4]. KOA is a very common subtype of osteoarthritis, and currently, more than 10% of the population worldwide has KOA [5], and the overall prevalence of KOA in China reaches 18% [6], and its prevalence increases with age. The main clinical manifestations of KOA are swelling pain and deformity of the knee joint and bring a series of complications, which severely reduce the quality of life and impose a severe pain and economic burden on patients [7]. At present, there are no clear and effective therapies for the treatment of KOA, and the clinical practice is mainly aimed at controlling pain and improving patient’s life quality, with the main goals of treatment being physical, pharmacologic, and surgical therapy [8, 9], but physical therapy has some limitations [10], and pharmacologic therapy mainly involves the use of NSAIDs, Long term use of NSAIDs can lead to related adverse reactions [11], and surgical treatment entails faced higher surgical and anesthetic risks, especially in patients with severe underlying disease. Therefore, it is essential to seek treatment that is both safe and effective.

TCM treatment has many advantages, such as convenience, little side effects, easy promotion, and relatively little economic burden, which is easily acceptable to patients. According to the guidance of TCM theory, TCM often treats KOA with the main treatment methods of warming the meridians and dispersing cold, nourishing blood and unblocking the meridians. The Zhuifeng Tougu Capsule is made from 24 TCMs, including Monkshood, Radix Aconiti kusnezoffi, Rhizoma Cyperi, Ligusticum wallichii, and Herba Ephedrae, and has the beneficial influences of clearing meridians, dispelling wind and dampness, relieving pain and dispelling cold. It has been applied to various joint diseases [12, 13]. Previous studies have reported that Zhuifeng Tougu Capsules can effectively improve OA symptoms. However, these studies are usually small-scale, non-multicenter, and have a short duration [14, 15].

In order to understand Zhuifeng Tougu Capsule’s efficacy and safety in treating KOA with cold dampness obstruction syndrome further, we collected more high-quality trials to provide evidence of the treatment’s efficacy. In our study, 10 research centers participated and 215 study cases were included. An evidence-based medical approach was used to investigate whether Zhuifeng Tougu Capsules could rationally be used in clinical practice through large-scale, high-quality randomized controlled trials.

## Methods

### Trial design and participants

In this multicentre, randomized, double-blind, double dummy, positive drug parallel controlled trial, 215 patients were included in the trial from April 2020 to April 2021, which was jointly completed by 10 research centers with the First Affiliated Hospital of Tianjin University of Traditional Chinese Medicine as the main unit. This study has been approved by site’s ethics committee (No. TYLL2019 [Y] No.016) and registered in the China clinical trial registry (No. ChiCTR2000028750).

### Eligibility criteria

Participants meeting the following criteria will be included: meeting the 1995 American Society of Rheumatology (ACR) diagnostic criteria for KOA, with or without osteoarthritis in other parts; 40 years old ≤ age ≤ 70 years old, regardless of gender; Kellgren Lawrence grading 1–3 patients; 4 points ≤ VAS pain score ≤ 7 points; Patients who have not undergone surgical treatment (including arthroscopic surgery, plastic surgery, chondrocyte transplantation, etc.); Patients who have not received joint cavity injection treatment within 30 days; Patients who have taken non-steroidal anti-inflammatory drugs (NSAIDs) should be stopped for at least 7 days; Patients who have taken cartilage protective drugs such as glucosamine and chondroitin sulfate need to stop using them for at least 30 days; Voluntarily sign the informed consent form and agree to participate in all visits, examinations and treatments according to the requirements of the trial protocol; Meets the diagnostic criteria for cold dampness obstruction syndrome in TCM.

The diagnostic standard of cold dampness obstruction syndrome in TCM is formulated with reference to the Guide for Diagnosis and Treatment of Osteoarthritis Combined with Disease and Syndrome (2019 version), which is specifically as follows:

Main symptoms: ① joint cold pain or swelling; ② The pain was fixed and aggravated in cold.

Secondary symptoms: ① cold and heavy limbs; ② Afraid of cold and fond of warmth; ③ Loose or clear stools.

Tongue and pulse: the tongue is light, the coating is white and greasy, and the pulse string is tight or slow.

There are two main symptoms; or one main symptom and two secondary symptoms, which can be diagnosed in combination with tongue and pulse.

### Exclusion criteria

Potential subjects who met the inclusion criteria were excluded if they fulfilled any of the following: Patients with rheumatoid arthritis, psoriatic arthritis, ankylosing spondylitis, gouty arthritis and other inflammatory arthritis diseases; Patients with severe and unstable cardiovascular and cerebrovascular diseases, acute and uncontrollable diseases, chronic diffuse connective tissue disease, severe hypertension (blood pressure > 160/100mmhg) or diabetes mellitus (fasting blood glucose > 11.1 mmol/l) not effectively controlled after treatment; Patients with active peptic ulcer or ulcer complicated with bleeding and perforation within the past year; Patients with active liver disease or abnormal liver function (ALT or AST ≥ upper limit of normal value); Abnormal renal function (SCR ≥ upper limit of normal value); Those who are allergic to test drug ingredients; Pregnant, prepared pregnant or lactating women; Those who are participating in other clinical trials; Those who have mental disease, have no insight, cannot express accurately or cannot take medicine on time, and cannot cooperate to complete the test.

### Discontinued

Serious safety issues occur in the trial, and investigators should promptly discontinue the trial when they believe that subject equity may be compromised; Significant lapses in clinical trial protocols were identified, or important deviations occurred in implementation, making it difficult to evaluate drug efficacy, safety; Discontinuation required by the sponsor (e.g., reasons for funding, administration, etc.); Withdrawal of the trial by the State Drug Administration, etc.

### Exclusion

Those who did not take trial medication or had a record of not taking trial medication after inclusion; None of the evaluable recorders after medication.

### Interventions

Experimental group: Zhuifeng Tougu Capsules (Tiandi Hengyi Pharmaceutical Co., Ltd., specification: 0.26 g/capsule, lot number: 191101), orally, four capsules twice a day; Glucosamine sulfate capsule mimetic, orally, two capsules three times a day; For 12 weeks; Control group: Glucosamine Sulfate Capsules (trade name: Yisuojia, Zhejiang Haizheng Pharmaceutical Co., Ltd., specification: 0.25 g/capsule, lot number: 71901101), orally, two capsules three times a day; Zhuifeng Tougu Capsule Simulator, orally, four capsules twice a day; For 12 weeks; Emergency medication: Voltalren capsule, oral, one tablet (75 mg) twice a day.

### Outcome

#### Primary outcome measure

The difference between WOMAC pain score and baseline at the end of the 12th week of medication: the University of Western Ontario and McMaster University (WOMAC) osteoarthritis index [16] mainly evaluates the structure and function of the knee joint from three aspects: pain, stiffness, and joint function. There are 24 items in total, including the basic symptoms and signs of osteoarthritis, including 5 items in the pain part, 2 items in the stiffness part. There are 17 items in the part of joint function. The main outcome measures of this study included the knee pain subscale score, and the remaining subscale scores were used as secondary outcome measures.

#### Secondary outcome measures

VAS pain scores [17]: To compare the intra group changes in knee VAS scores from baseline after 12 weeks of medication, between group differences in change values. Baseline period data were obtained by subject review of the condition for 1 week prior to enrollment, and visit period data were obtained by subject review of the condition for 4 weeks prior to enrollment.

Self-condition assessment VAS score [18]: After 12 weeks of treatment, the changes within the group and the differences between the groups in the VAS score of their overall condition compared with the baseline period were compared. Baseline period data were obtained by subject review of the condition for 1 week prior to enrollment, and visit period data were obtained by subject review of the condition for 4 weeks prior to enrollment.

WOMAC KOA score [19]: To compare the intra group changes in WOMAC KOA score from baseline after 12 weeks of medication, between group differences in change values. The effects of the test drugs on knee structure and function were evaluated in three dimensions: pain, stiffness, and joint motor function.

TCM syndrome score and TCM syndrome efficacy: The scoring of TCM syndromes is based on the "Guiding Principles for Clinical Research of New Traditional Chinese Medicine" (2002 edition) published by China Medical Science and Technology Press. Evaluation criteria for the efficacy of TCM syndrome: recovery: symptom score reduction ≥ 90%; Significant effect: 70% ≤ symptom score reduction < 90%; Effective: 30% ≤ symptom score reduction < 70%; Invalid: Symptom points reduced by < 30%.

Erythrocyte sedimentation rate (ESR): the intragroup change in serum ESR levels compared with the baseline period after 12 weeks of comparative medication, and the change values differed between groups. Baseline period data were obtained by subject enrollment visit testing, and visit period data were obtained by subject visit testing.

C-reactive protein (CRP): the intragroup change in serum CRP levels compared with the baseline period after 12 weeks of treatment, with between group differences in change values. Baseline period data were obtained by subject enrollment visit testing, and visit period data were obtained by subject visit testing.

Knee suprapatellar bursa effusion depth: To compare the intragroup change in knee suprapatellar bursa effusion depth from baseline after 12 weeks of medication, between group differences in change values. The baseline phase data were obtained by the subject's enrollment visit knee ultrasound test, and the visit data were obtained by the subject's enrollment visit knee ultrasound test.

Emergency drug use: the total amount of Voltalren capsules used between both groups after treatment was compared. The total amount of Voltalren capsules used during the treatment was obtained according to the records of the subject's diary card.

Adverse event (AE): means all adverse medical event occurring after a subject has received a drug product for test use that can manifest as symptomatic signs, illness, or abnormal laboratory tests but is not necessarily causally related to the drug product used for the test.

### Statistical analysis

The full analysis set (FAS) and per protocol set (PPS) were used to analyze the all of outcome measures. Measurement data: t-test, paired t-test, rank sum test, paired rank sum test and other methods were used; The corrected chi square test and Fisher exact test were used for counting data; Rank data: rank sum test was used; PP analysis and ITT analysis were carried out for the overall evaluation indicators and main efficacy indicators. A two-tailed level of 0.05 was considered as having statistical significance. All data were analyzed using SAS (version 9.1.4).

## Results

### Case collection and completion

**Fig. 1:**
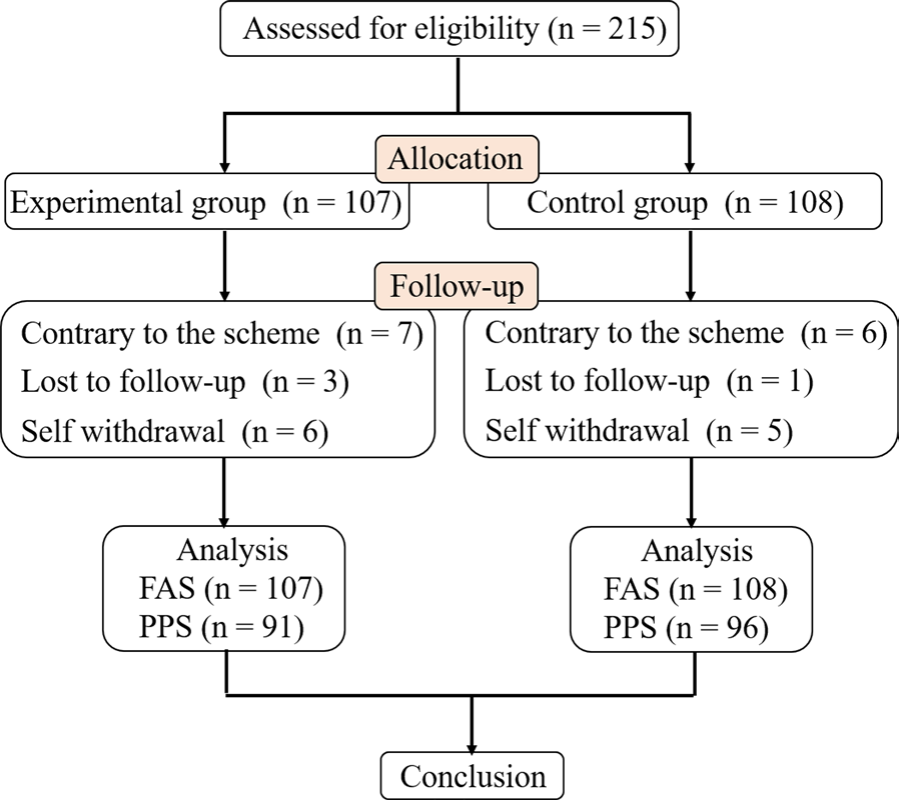
Systematic illustration of study design

According to the registration scheme, this study is a multicenter clinical study, involving a total of 10 research centers. A total of 215 study cases were included from April 2020 to April 2021. The clinical symptom indicators (WOMAC pain score, VAS pain score, Self-condition assessment VAS score, WOMAC knee osteoarthritis score, TCM syndrome score and TCM syndrome efficacy) were measured. 187 subjects completed the 12 week experiment, including 91 in the experimental group and 96 in the simulator group (Fig. 1).

### Analysis of baseline data

In Table 1, we did not find any significant differences (*P* > 0.05) between both groups in age, gender, nationality, occupation, height and weight. No significant differences (*P* > 0.05) between both groups were found in basic vital signs (such as temperature, heart rate, blood pressure, respiration).No significant differences (*P *> 0.05) between both groups were found in the diagnosis of knee osteoarthritis, X-ray grading, diagnosis of cold dampness obstruction syndrome, marriage and childbirth history, allergy history, family history, smoking status, drinking status, comorbidities, past medical history, and previous medication (*P* > 0.05). No significant difference (*P* > 0.05) between both groups were found in the effectiveness evaluation indicators (VAS pain score, WOMAC KOA score, number of painful joints, number of swollen joints, Self-condition assessment VAS score, TCM syndrome score, TCM single symptom score) and other indicators, indicating that both groups were comparable at baseline.

**Table 1:** Basic characteristics of patients with knee osteoarthritis (x ® ± s/N (%))

	Characteristics		Experimental group	Control group	*P* value
FAS	Gender	Male	27 (25.47)	17 (15.74)	0.078
		Female	79 (74.53)	91 (84.26)	
	Age (years)		58.04 ± 7.13	58.03 ± 7.39	0.942
	Occupation	Manual	15 (38.46)	17 (42.50)	0.715
		Nonmanual	24 (61.54)	23 (57.50)	
	Weight (kg)		66.18 ± 11.93	65.92 ± 12.79	0.696
	Height (cm)		161.54 ± 7.24	161.57 ± 7.10	0.894
PPS	Gender	Male	22 (24.18)	12 (12.50)	0.039
		Female	69 (75.82)	84 (87.50)	
	Age (years)		57.91 ± 7.05	58.38 ± 7.31	0.652
	Occupation	Manual	14 (36.84)	16 (42.11)	0.639
		Nonmanual	24 (63.16)	22 (57.89)	
	Weight (kg)		66.35 ± 12.49	65.93 ± 13.21	0.697
	Height (cm)		161.48 ± 7.42	161.19 ± 7.16	0.694

### Primary outcome

#### WOMAC pain score

**Fig. 2:**
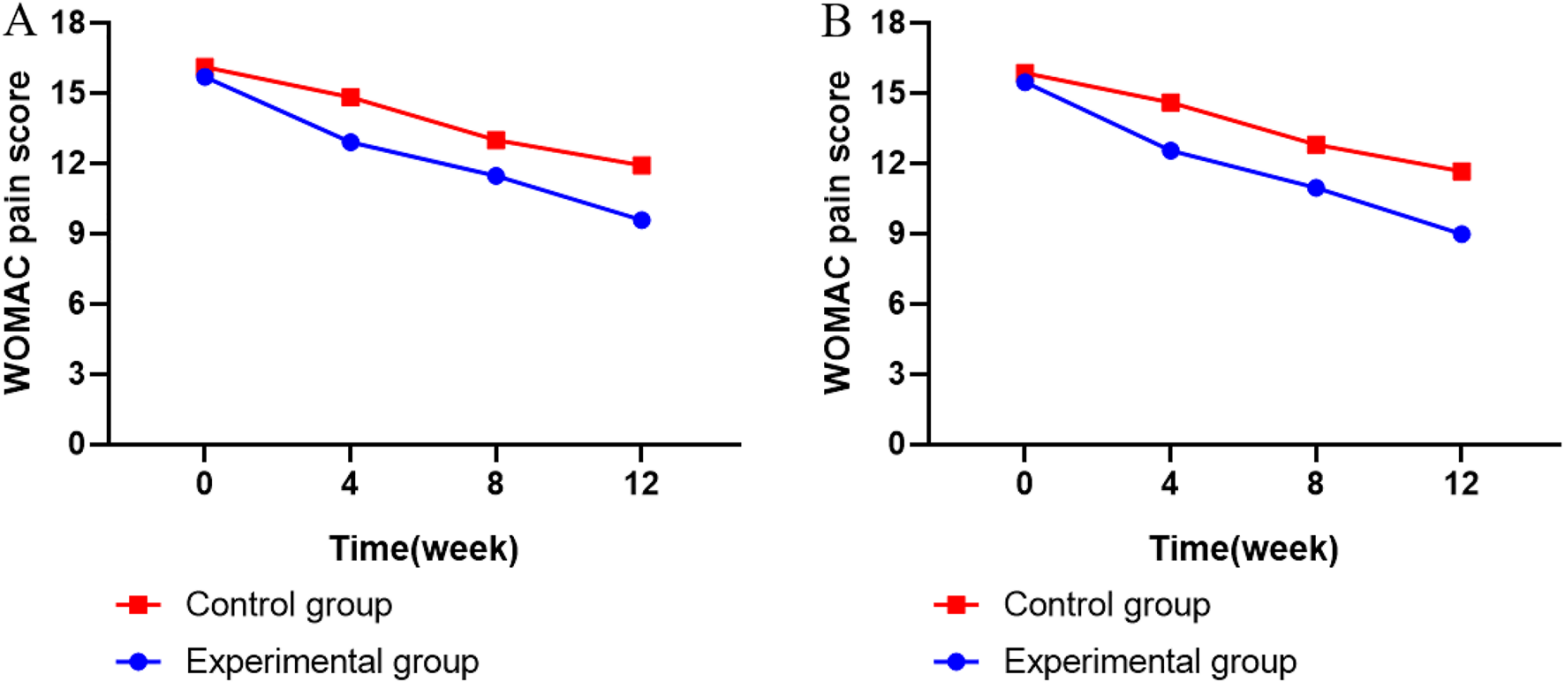
Changes in WOMAC pain scores between two groups. **A**. (FAS) **B**. (PPS)

In Table 2, for comparative analysis, we did not find a significant difference in WOMAC pain score between both groups of patients before treatment (*P* > 0.05). After treatment, WOMAC pain scores in both groups of patients decreased compared to before treatment (*P* < 0.01), and the decrease in the intervention group was more significant compared to control (*P* < 0.01). The WOMAC pain score decreased following the change of time (Fig. 2).

**Table 2:** Changes in WOMAC pain scores between two groups (x ® ± s)

	Group	N	Before treatment	After treatment	*P* value
FAS	Experimental group	107 (0)	15.70 ± 6.48	9.58 ± 5.19	< 0.01
	Control group	108 (0)	16.11 ± 6.23	11.91 ± 5.58	< 0.01
	Comparison between groups	*P* value	0.572	< 0.01	
PPS	Experimental group	91 (0)	15.47 ± 6.16	8.97 ± 4.71	< 0.01
	Control group	96 (0)	15.86 ± 5.82	11.66 ± 5.45	< 0.01
	Comparison between groups	*P* value	0.547	< 0.01

### Secondary outcome

#### VAS pain score

In FAS and PPS (Table 3), we did not detect significant difference in VAS pain score between both groups before treatment (*P* > 0.05), which was comparable. After treatment, the pain score of both groups of patients decreased compared to before treatment (*P* < 0.01), and the pain score of the experimental group decreased more significant compared to control (*P* < 0.01).

**Table 3:** Changes in VAS scores for pain between the two groups (x ® ± s)

	Group	N	Before treatment	After treatment	*P* value
FAS	Experimental group	100 (7)	5.46 ± 0.91	2.54 ± 1.22	< 0.01
	Control group	104 (4)	5.37 ± 1.05	3.76 ± 1.23	< 0.01
	Comparison between groups	*P* value	0.441	< 0.01	
PPS	Experimental group	91 (0)	5.41 ± 0.92	2.55 ± 1.22	< 0.01
	Control group	96 (0)	5.33 ± 1.02	3.72 ± 1.20	< 0.01
	Comparison between groups	*P* value	0.519	< 0.01	

#### Self condition assessment VAS score

In the FAS and PPS (Table 4), we did not found statistically significant difference in VAS score of self-condition assessment between both groups of patients before treatment (*P* > 0.05), which was comparable. After treatment, the self-condition assessment VAS score of both groups decreased compared to baseline (*P* < 0.01), and the score of the intervention group decreased more significant compared to control (*P* < 0.01).

**Table 4:** Self condition assessment VAS score (x ® ± s)

	Group	N	Before treatment	After treatment	*P* value
FAS	Experimental group	107 (0)	5.28 ± 1.05	2.48 ± 1.22	< 0.01
	Control group	108 (0)	5.01 ± 1.06	3.48 ± 1.36	< 0.01
	Comparison between groups	*P* value	0.088	< 0.01	
PPS	Experimental group	91 (0)	5.23 ± 1.05	2.49 ± 1.23	< 0.01
	Control group	96 (0)	5.00 ± 1.06	3.48 ± 1.35	< 0.01
	Comparison between groups	*P* value	0.168	< 0.01	

#### WOMAC KOA score

In FAS and PPS (Table 5), no significant difference was found in WOMAC KOA score between both groups of patients before treatment (*P* > 0.05), which was comparable. After treatment, the WOMAC KOA score of both groups decreased compared to baseline (*P* < 0.01), and the arthritis score of the intervention group decreased more significant compared to control (*P* < 0.01).

**Table 5:** WOMAC knee osteoarthritis score (x ® ± s)

	Group	N	Before treatment	After treatment	*P* value
FAS	Experimental group	107 (0)	73.79 ± 30.99	31.24 ± 19.59	< 0.01
	Control group	108 (0)	76.88 ± 30.07	21.27 ± 20.92	< 0.01
	Comparison between groups	*P* value	0.445	< 0.01	
PPS	Experimental group	91 (0)	72.05 ± 29.24	39.12 ± 21.80	< 0.01
	Control group	96 (0)	75.48 ± 27.43	54.42 ± 27.17	< 0.01
	Comparison between groups	*P* value	0.395	< 0.01	

#### TCM syndrome score

In FAS and PPS (Table 6), no significant difference was detected in TCM syndrome scores between both groups of patients before treatment (*P* > 0.05). After treatment, the TCM syndrome score of both groups decreased compared to before treatment (*P* < 0.01), and the TCM syndrome score of the intervention group decreased more significant compare to control (*P* < 0.01).

**Table 6:** TCM Syndrome Score (x ® ± s)

	Group	N	Before treatment	After treatment	*P* value
FAS	Experimental group	107 (0)	10.51 ± 2.90	6.37 ± 2.62	< 0.01
	Control group	108 (0)	10.66 ± 2.84	7.69 ± 2.98	< 0.01
	Comparison between groups	*P* value	0.679	< 0.01	
PPS	Experimental group	91 (0)	10.36 ± 2.82	6.04 ± 2.45	< 0.01
	Control group	96 (0)	10.61 ± 2.86	7.63 ± 3.01	< 0.01
	Comparison between groups	*P* value	0.690	< 0.01	

#### TCM single item syndrome score

The single TCM syndromes were recorded and scored. In FAS and PPS (Table 7), We did not find a significant difference in the single TCM syndromes scores (patients' self-rated pain score, morning stiffness, joint cold pain) between both groups of patients before treatment (*P* > 0.05). After treatment, the patients’ self-rated pain scores decreased compared to before treatment (*P* < 0.01), and the self-rated pain scores of the experimental group decreased more significant compared to control (*P* < 0.01).The morning stiffness of the patients after treatment decreased compared to before treatment, and the self-rated pain score of the experimental group decreased more significant compared to control in FAS (*P* < 0.05) and PPS (*P* < 0.01). In FAS and PPS, the joint cold pain of patients after treatment decreased compared to baseline (*P* < 0.05), and the self-rated pain score of the experimental group decreased more significant compared to baseline (*P* < 0.01).

**Table 7:** TCM single item syndrome score (x ® ± s)

Symptom		FAS			PPS		
		Experience group (107)	Control group (108)	*P* value	Experimental group (91)	Control group (96)	*P* value
Pain	Before treatment	3.11 ± 0.37	3.11 ± 0.37	0.984	3.10 ± 0.37	3.13 ± 0.36	0.638
	After treatment	2.24 ± 0.53	2.54 ± 0.52	< 0.01	2.15 ± 0.47	2.52 ± 0.52	< 0.01
	*P* value	< 0.01	< 0.01		< 0.01	< 0.01	
Morning stiffness	Before treatment	1.89 ± 0.68	1.92 ± 0.74	0.811	1.85 ± 0.68	1.88 ± 0.73	0.823
	After treatment	1.45 ± 0.54	1.67 ± 0.63	0.011	1.38 ± 0.51	1.65 ± 0.63	< 0.01
	*P* value	< 0.01	< 0.01		< 0.01	< 0.01	
Joint cold pain	Before treatment	1.94 ± 0.23	1.95 ± 0.21	0.748	1.93 ± 0.25	1.96 ± 0.20	0.464
	After treatment	1.64 ± 0.48	1.76 ± 0.43	0.049	1.62 ± 0.49	1.77 ± 0.42	0.021
	*P* value	< 0.01	< 0.01		< 0.01	< 0.01	

#### Comparison of TCM effectiveness rates

In Table 8, after treatment, the total effective rate of TCM syndrome efficacy in the experimental group was 67.03%, while that in the control group was 47.92%. The total effective rate of the experimental group was increased in both FAS (*P* < 0.05) and PPS (*P* < 0.01) analysis compared with control.

**Table 8:** Comparison of TCM effectiveness rates (N/%)

Curative effect index	FAS		PPS	
	Experimental group (107)	Control group (108)	Experimental group (91)	Control group (96)
Markedly effective	8 (7.48)	3 (2.78)	7 (7.69)	3 (3.13)
Effective	60 (56.07)	47 (43.52)	54 (59.34)	43 (44.79)
Ineffective	39 (36.45)	58 (53.70)	30 (32.97)	50 (52.08)
Total effective rate	63.55	46.30	67.03	47.92
*P* value	0.011		0.008	

### Laboratory index

In FAS and PPS (Tables 9, 10, 11), after treatment, no significant difference was detected in ESR level, CRP level and suprapatellar bursa effusion depth between both groups compared to baseline (*P* > 0.05).

**Table 9:** Comparison of ESR levels between the two groups (x ® ± s)

	Group	N	Before treatment	After treatment	*P* value
FAS	Experimental group	106 (1)	11.76 ± 8.72	11.69 ± 8.50	0.774
	Control group	107 (1)	12.91 ± 8.59	12.34 ± 8.74	0.363
	Comparison between groups	*P* value	0.141	0.634	
PPS	Experimental group	91 (0)	11.92 ± 9.07	11.51 ± 8.47	0.595
	Control group	95 (1)	13.05 ± 8.62	12.34 ± 8.92	0.425
	Comparison between groups	*P* value	0.202	0.550

**Table 10:** Comparison of CRP levels between the two groups (x ® ± s)

	Group	N	Before treatment	After treatment	*P* value
FAS	Experimental group	74 (33)	2.79 ± 3.22	2.89 ± 3.48	0.681
	Control group	79 (29)	3.27 ± 4.75	3.04 ± 4.31	0.459
	Comparison between groups	*P* value	0.813	0.723	
PPS	Experimental group	64 (27)	2.77 ± 3.33	2.81 ± 3.51	0.638
	Control group	71 (25)	3.45 ± 4.97	3.14 ± 4.38	0.451
	Comparison between groups	*P* value	0.632	0.438

**Table 11:** Suprapatellar bursa effusion depth (x ® ± s)

	Group	N	Before treatment	After treatment	*P* value
Left	Experimental group	56 (0)	4.34 ± 2.49	4.25 ± 7.18	0.928
	Control group	60 (0)	4.86 ± 2.33	4.35 ± 4.95	0.411
	Comparison between groups	*P* value	0.141	0.450	
Right	Experimental group	53 (0)	4.11 ± 2.02	3.93 ± 6.57	0.841
	Control group	58 (0)	4.85 ± 3.34	4.06 ± 5.41	0.212
	Comparison between groups	*P* value	0.446	0.903	

### Emergency medication usage

During the study period, only one subject in the experimental group used emergency medication, and there was a lack of statistically significant distinction observed among the groups.

### Adverse events

In Table 12, a total of 32 adverse events occurred in this clinical study, of which 15 adverse events occurred in subjects of the experimental group with an adverse event rate of 14.02% and 17 adverse events occurred in subjects of the control group with an adverse event rate of 15.74%, no statistically significant difference was showed in the occurrence of adverse events between both groups (*P* > 0.05).

**Table 12:** Analysis of adverse events

	Experimental group	Control group	*P* value	
N (miss)	107 (0)	108 (0)		
Adverse events	15 (14.02%)	17 (15.74%)	0.134	0.714

Adverse reactions occurred in 2 cases (4 times) in the experimental group, with an incidence of 1.87%, which were subject 98 (gastrointestinal discomfort, which was judged by the investigator to be possibly related to the study drug) and subject 112 (abnormal liver function indicators, which was judged by the investigator to be possibly related to the study drug). The severity of adverse reactions in 2 subjects was mild, and the dosage of study drug was not adjusted or other symptomatic treatment methods were taken in the research process. Adverse reactions occurred in 5 cases (7 times) in the control group, with an incidence of 4.63%. Among them, No. 36 (mild adverse reaction, abnormal liver function, investigator's judgment is likely to be associated with the study drug), No. 10 (moderate adverse reaction, gastric ulcer, investigator's judgment may be associated with the study drug), No. 15 (mild adverse reaction, abnormal ECG, investigator's judgment may be associated with the study drug), No. 25 (mild adverse reaction, abnormal liver function, investigator's judgment may be related to the study drug) No. 120 (mild adverse reaction, abnormal liver function, which may be associated with the study drug according to the judgment of the investigator), the dosage of the study drug was not adjusted or other symptomatic treatment methods were adopted during the study, and the results are shown in Table 13. There were no serious adverse events, serious adverse reactions and deaths in the study.

**Table 13:** Occurrence of adverse events in various systems

Adverse events	Experimental group			Control group		
	Cases	Case times	Incidence	Cases	Case times	Incidence
Digestive system	2	4	1.87%	4	6	3.7%
Circulatory system	0	0	0%	1	1	0.93%

## Discussion

At present, personalized and tiered treatment plans are advocated for the treatment of KOA, and drug therapy is still the main treatment method for KOA [20]. The Western medicine for KOA mainly employs NSAIDs, glucosamine hydrochloride, glucocorticoids, intra-articular injections of relevant drugs, or joint replacement for the purpose of relieving pain and improving joint function. Although pain relief is short-term, the efficacy of these drugs is inconsistent and all have varying degrees of adverse effects [21]. Therefore, it is essential to determine a safer, stable and effective treatment plan for improving the long-term prognosis and life’s quality of patients with KOA. Recently, TCM has gained attention in the treatment of KOA, and reports have indicated that the TCM formula has a superior safety profile and can effectively shorten the treatment time and improve the efficacy of patient treatment. Here, we observe the efficacy of Zhuifeng Tougu Capsules through high-quality and large-scale randomized controlled trials.

This RCT is the largest, multi center, and longest lasting study to investigate the effect of Zhuifeng Tougu Capsule on knee joint pain to date. Pain is the main symptom of KOA, and effective control of joint pain in patients is crucial for improving their quality of life. Accordingly, the main clinical indicator was the improvement in pain among patients in this study. After 12 weeks of treatment, WOMAC pain scores of subjects in both groups were significantly lower compared to baseline, indicating that both treatments can effectively reduce the pain of patients. There was a statistical significance between the two groups in terms of pain scores, suggesting that the analgesic effect of Zhuifeng Tougu Capsule is better than Glucosamine Sulfate Capsule. The results of secondary clinical indicators indicated that the VAS pain score, Self-condition assessment VAS score, WOMAC KOA score, TCM syndrome score, and TCM syndrome efficacy in both groups of subjects decreased compared to before treatment, indicating that both treatments can significantly reduce patient symptoms and improve knee joint function, while the intervention group showed significantly lower scores in all aspects than control, It is suggested that the effect of Zhuifeng Tougu Capsules on improving knee joint function is better than that of Glucosamine Sulfate Capsules. Safety evaluations showed no serious adverse events, serious adverse reactions, or deaths as a result of the study. In the experimental group, adverse events occurred at a rate of 14.02%, and adverse reactions at a rate of 1.87%, while in the control group, adverse events occurred at a rate of 15.74%, and adverse reactions at a rate of 4.63%. No significant difference was found in adverse event’s incidence between both groups of subjects, and we did not adjust the dosage of study drugs or adopt other targeted treatment methods for the subjects who experienced adverse events during the research process. This suggests a good safety profile for Zhuifeng Tougu Capsules.

TCM formula has the characteristics of multi-component, multi-target and multi-level in the treatment of OA. Zhuifeng Tougu Capsule has the effects of clearing meridians, dispelling wind and dampness, relieving pain and dispelling cold, and is a good prescription for treating cold dampness obstruction syndrome. It is based on Xiaohuoluo Dan, Lingguizhugan Decoction and Jiuwei Qianghuo Decoction. It is composed of 24 traditional Chinese medicinal materials, including Radix Aconiti, Rhizoma Cyperi, Herba Ephedrae, Radix Aconiti kusnezoffi, Gentiana macrophyllae, Angelica sinensis, Red adzuki bean, Ligusticum wallichii, Notopterygium Notopterygii, Radix Angelicae dahuricae, Radix Glycyrrhizae, Rhizoma Atractylodis Macrocephalae, Myrrh, Frankincense, Earthworm, Monkshood, Poria cocos, Ramulus Cinnamomi, Rhizoma Gastrodia elata, Radix Glycyrrhizae, Radix Saposhnikoviae, and Cinnabar, etc. According to Fingerprint Analysis and Ultra Performance Liquid Chromatography, the effective ingredients of Zhuifeng Tougu Capsule are mainly from Ligusticum chuanxiong, Radix Aconiti kusnezoffii, Gentiana macrophylla, notopterygium Notopterygii, radix paeoniae rubra, Radix Glycyrrhizae and Radix Saposhnikoviae [22]. Experimental studies have shown that Zhuifeng Tougu Capsule and glucosamine sulfate can effectively inhibit the activation of TLR4/MyD88/NF-κB signaling pathways in articular cartilage and reduce inflammatory factor production, thereby delaying the progression of KOA [22]. Modern pharmacological research also shows that Zhuifeng Tougu Capsule has pharmacological effects of anti-inflammatory, analgesic, improving blood circulation and reducing blood viscosity, so as to regulate the immune function of the body [23]. In the formula, Monkshood [24, 25], Angelica sinensis [26, 27], Red peony[28, 29], Asarum [30, 31], Radix Angelicae dahuricae [32], Rhizoma Gastrodia elata [33], Atractylodes macrocephala [34], Herba Ephedrae [35], Ramulus Cinnamomi [36], Gastrodia elata [37], and Radix Saposhnikoviae [38] have anti-inflammatory and analgesic effects, while Herba Ephedrae [39], Radix Aconiti kusnezoffi [40], and Gentiana macrophylla [41] can exert analgesic effects by regulating the central nervous system. In addition, the terpenoids in Rhizoma Cyperi can reduce chondrocyte inflammation and extracellular matrix degradation, improve mouse osteoarthritis [42], while Ligusticum wallichii can affect chondrocytes, synoviocytes and other cells, which is closely related to the occurrence and development of OA, and effectively treat osteoarthritis through multi-target and multi-channel properties [43, 44]. Therefore, Zhuifeng Tougu Capsule can improve and alleviate patient symptoms, improve knee joint function, and play a unique role in pain relief.

However, our research still has some limitations. Firstly, the difficulties of the study include timely treatment of subjects, poor compliance, loss of follow-up due to medication violations, and accuracy of pain records during the study process. Secondly, we only used commonly effective indicators in our study, and the results of laboratory anti-inflammatory indicators were not ideal and relevant imaging data were not obtained.

## Conclusions

Zhuifeng Tougu capsule can treat knee osteoarthritis with cold dampness obstruction syndrome, improve morning stiffness, joint cold pain and other symptoms and improve knee joint mobility function, especially in relieving pain, and has a good safety profile.

## Data Availability

Data and materials presented in this study are available on request from the corresponding author.
